# Classification and Current Management of Inner Ear Malformations

**DOI:** 10.4274/balkanmedj.2017.0367

**Published:** 2017-09-29

**Authors:** Levent Sennaroğlu, Münir Demir Bajin

**Affiliations:** 1 Department of Otolaryngology, Hacettepe University School of Medicine, Ankara, Turkey

**Keywords:** Inner ear malformations, cochleovestibular malformations, classification, treatment, incomplete partition, cochlear hypoplasia, Radiology, Surgery

## Abstract

Morphologically congenital sensorineural hearing loss can be investigated under two categories. The majority of congenital hearing loss causes (80%) are membranous malformations. Here, the pathology involves inner ear hair cells. There is no gross bony abnormality and, therefore, in these cases high-resolution computerized tomography and magnetic resonance imaging of the temporal bone reveal normal findings. The remaining 20% have various malformations involving the bony labyrinth and, therefore, can be radiologically demonstrated by computerized tomography and magnetic resonance imaging. The latter group involves surgical challenges as well as problems in decision-making. Some cases may be managed by a hearing aid, others need cochlear implantation, and some cases are candidates for an auditory brainstem implantation (ABI). During cochlear implantation, there may be facial nerve abnormalities, cerebrospinal fluid leakage, electrode misplacement or difficulty in finding the cochlea itself. During surgery for inner ear malformations, the surgeon must be ready to modify the surgical approach or choose special electrodes for surgery. In the present review article, inner ear malformations are classified according to the differences observed in the cochlea. Hearing and language outcomes after various implantation methods are closely related to the status of the cochlear nerve, and a practical classification of the cochlear nerve deficiency is also provided.

Inner ear malformations (IEM) represent approximately 20% of congenital hearing loss cases based on radiology (1,2). Majority of these patients have bilateral severe to profound hearing loss and are candidates for cochlear implantation (CI). Those cases with severe malformations may require different surgical approach for implant placement. Decision making between CI and auditory brainstem implantation (ABI) may also be challenging in some cases of IEMs.

It is very important to classify IEMs correctly and have a universally accepted system. There is a large variety of malformations which complicate diagnosis and management. Proper classification is as important as using a common language. If we do not have a common language, it is very difficult to understand the findings of another research group. Universally accepted classification of IEMs is particularly important in the field of CI. Surgeons, audiologists, speech and language specialists should be familiar with this system; otherwise, it will be very difficult to understand and compare the outcome after CI surgery in this particular patient group.

There are certain challenges in the management of IEMs:

1. Cerebrospinal fluid gusher and risk for meningitis

2. Facial nerve anomalies

3. Decision making for the surgical approach and the type of electrode

4. Choosing the correct implantation method; CI vs ABI

5. Timing of surgery

Classification of IEMs is based on differences in cochlear anatomy in various malformations. With this classification, cochlear anomalies with similar appearance are grouped together. They demonstrate similar clinical findings, and treatment options. This may not represent the functional outcome with CI, which is closely related to the situation of the cochlear nerve. If there is cochlear nerve deficiency, this will have a negative influence on the audiological and speech and language developmental after implantation. Therefore, during preoperative decision making for choosing the method of implantation, three factors should be considered: classification of IEMs, situation of the cochlear nerve and preoperative audiological findings. Only in this way, a clinician may have a better estimate of the audiological outcome in a given IEM. This is very important during preoperative counseling of the family. In the present paper, classification of cochlear nerve abnormalities is also provided.

This review article describes the characteristics of anomalies in eight groups together with treatment options.

## Normal Cochlea

It is important to become familiar with normal anatomy of the cochlea as seen on high resolution computerized tomography of the temporal bone. Temporal bone HRCT was performed on sections of 0.5 mm in thickness. The use of a 1.5- or 3-T MR imaging system is preferred for inner ear examinations, and it is strongly suggested to be done under general anesthesia. A thin-section gradient-echo sequence that is heavily T2 weighted is best suited for evaluation of the fluid-filled spaces of the membranous labyrinth and the eighth cranial nerve. A section thickness of as little as 0.4-0.7 mm is preferred for optimal delineation and to allow the generation of high-quality multiplanar reformatted images. Oblique sagittal reformatted images are obtained in planes perpendicular to the course of the seventh and eighth nerves in the internal auditory canal (IAC) and cerebellopontine angle. Routine axial T2-weighted imaging of the brain should be performed in all patients to exclude central nervous system causes of sensorineural hearing loss.

Cochlea has 2½ or 2¾ turns (Figure 1). Mid-modiolar view is the most important section to evaluate internal architecture of cochlea and differentiate normal cochlea and incomplete partition anomalies. Mid-modiolar view (Figure 1a) demonstrates the modiolus as a quadrangular or pentagonal structure in the center of the basal turn and between the basal and middle turns of the cochlea (2). Interscalar septa are thicker partitions between the inner wall of the cochlea and the modiolus, which separate the normal cochlea into 2½ or 2¾ turns; the basal, middle and apical turns. The cochlear aperture (CA) (bony canal for cochlear nerve) is the central bony passage at the base of the modiolus transmitting the cochlear nerve and blood vessels.

Section inferior to the mid-modiolar view, passes through the area of the round window niche (Figure 1b). This section shows the basal, middle, and apical cochlear turns. Basal turn is in continuity at this section. It is important to see the interscalar septum between the middle and apical turns. This view is very important to differentiate cochlear hypoplasia (CH) type IV, in addition to incomplete partition type II. A thin-section, heavily T2 weighted image of the cochlea demonstrates fluid filled spaces of the cochlea (scala tympani and vestibuli), modiolus and cochlear nerve (Figure 1c).

## I-INNER EAR MALFORMATIONS

According to present literature (3,4), IEMs are classified into eight distinct groups. Characteristics of each group can be found in Table 2.

### 1- Complete Labyrinthine Aplasia (Michel Deformity)

**FIG. 2.**
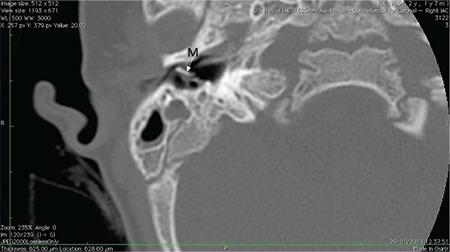
Complete labyrinthine aplasia; absence of cochlea, vestibule, semicircular canals, vestibular and cochlear aqueducts. Middle ear ossicles are usually present (M=malleus).

Complete labyrinthine aplasia (CLA) is the absence of the cochlea, vestibule, semicircular canals (SCCs), vestibular and cochlear aqueducts (Figure 2). The petrous bone may be hypoplastic whereas the otic capsule may be hypoplastic or aplastic (4). In the majority of patients, the IAC consists only of the facial canal and the labyrinthine, tympanic and mastoid segments of the facial nerve can be identified in the temporal bone. In some patients, however, it may not be possible to observe the facial canal in the temporal bone in spite of normal facial functions. Development of middle ear ossicles are usually normal.

According to radiological findings (4), three subgroups of CLA are present:

### a- CLA with hypoplastic or aplastic petrous bone

In these cases CLA is accompanied by hypoplasia or aplasia of the petrous bone. Middle ear may be adjacent to posterior fossa.

### b- CLA without otic capsule 

In this group of CLA, formation of the petrous bone is normal, but the otic capsule is hypoplastic or aplastic.

### c- CLA with otic capsule

Formation of the petrous bone and the otic capsule is normal. Only in this group of CLA with otic capsule development, labyrinthine segment of the facial canal is in its normal location. This shows that otic capsule formation is essential for the facial canal to obtain its normal position.

### Audiological Findings

These patients either do not show a response during audiological evaluation or they may demonstrate profound sensorineural hearing loss on low frequencies which should be accepted as vibrotactile stimulation.

### Management

It is not possible to perform CI surgery in these children as there is no inner ear development. In the first Consensus Meeting on ABI in Children (5), CLA together with other severe IEMs are accepted as “Definite Indications for ABI” (Table 1). ABI is the only surgical option for hearing habilitation.

### 2- Rudimentary Otocyst

**FIG. 3.**
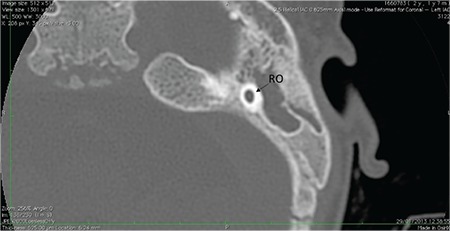
Rudimentary otocyst (RO); incomplete millimetric representations of the otic capsule (round or ovoid in shape) without an IAC.

A rudimentary otocyst is used to define incomplete milimetric representations of the otic capsule (round or ovoid in shape) without an IAC (Figure 3). Parts of the SCCs may accompany rudimentary otocyst. This pathology represents an anomaly between a Michel deformity and common cavity (CC). In Michel deformity, there is no inner ear development, while in CC, there is an ovoid or round cystic space instead of a separate cochlea and vestibule. The CC communicates with the brainstem via the nerves in the IAC. The rudimentary otocyst is a few millimeters in size without the formation of an IAC.

### Audiological Findings

Similar to CLA, either there is no response at all or profound loss on low frequency which is vibrotactile stimulation.

### Management

The fact that there is no connection between the otocyst and the brainstem is a contraindication to CI surgery. Rudimentary otocyst is also a definite indication for ABI (Table 1).

### 3- Cochlear Aplasia

Cochlear aplasia is the absence of the cochlea. The labyrinthine segment of the facial nerve is anteriorly displaced and occupies the normal location of the cochlea. Vestibule and SCCs are in their normal anatomic location; at the posterolateral part of IAC.

There are two subgroups according to accompanying vestibular system:

**a- Cochlear aplasia with normal labyrinth (Figure 4a):** Vestibule and SCCs are normally developed.

**b- Cochlear aplasia with a dilated vestibule (CADV) (Figure 4b):** Vestibule and SCCs show dilatation. It is very important to differentiate CADV from a common cavity (CC) deformity. In the latter IAC is normally developed, and dilated vestibule occupies normal location at the posterolateral part of the fundus. CI surgery should not be done in CADV. In CC, however, IAC is usually posteriorly directed and opens into the center of CC. If cochleovestibular nerve (CVN) is present, CI can be done in CC. However, in some patients, it may be very difficult to distinguish between these entities. As in every CI candidate, during preoperative evaluation for implantation, audiological findings should be taken into account in choosing the right method of implantation.

Cochlear aplasia with normal labyrinth is usually symmetric. Similar appearance is present in different individuals, suggesting a genetic etiology. In CADV, however, asymmetric development may be present; pathology may be due to genetic or environmental factors. Otic capsule development is always normal.

### Audiological Findings

These patients do not have a hearing level, the only stimulation can be vibrotactile.

### Management

As there is no inner ear development, ABI is the only feasible surgical option to provide hearing in children with cochlear aplasia (5).

### 4- Common Cavity

**FIG. 5.**
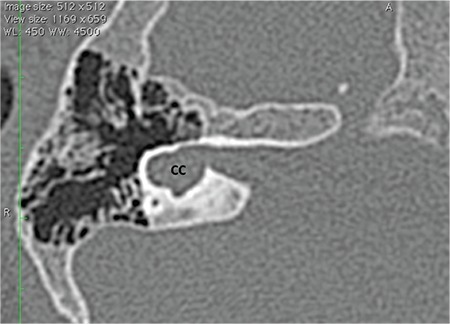
Common cavity (CC); a single, ovoid or round chamber, representing cochlea and vestibule.

A CC is defined as a single, ovoid or round chamber, representing cochlea and vestibule (Figure 5). Theoretically, this structure has cochlear and vestibular neural structures. There may be accompanying SCC or their rudimentary parts. The IAC usually enters the cavity at its center. Cases with vestibular dilatation are occasionally termed as “vestibular common cavity”; however, this is not a correct term.

CC needs to be differentiated from cochlear aplasia with dilated vestibule (1). CADV (Figure 4b) has a dilated vestibule and SCCs at the posterolateral part of the IAC fundus, which is their usual location. External outline resembles the normal labyrinth. The enlarged vestibule is at its expected location. The accompanying SCCs may be enlarged or normal. A CC (Figure 5), on the other hand, is an ovoid or round structure. SCC’s or their rudimentary parts may accompany a CC. The IAC usually enters the cavity at its center. The location of a CC may be anterior but usually posterior to the normal location of the labyrinth. It is very important to differentiate these malformations from each other, because CI in a CC may result in acoustic stimulation, whereas in CAVD, no functional stimulation will occur with CI. In spite of this, it may sometimes be difficult to differentiate between the two malformations.

Correct terminology for the nerve entering the CC is common CVN. CVN has to be demonstrated by 3 Tesla MRI in candidates undergoing evaluation for CI candidacy. Theoretically CVN contains cochlear and vestibular nerve fibers, however with the present radiological investigations, it is not possible to determine the percentage of cochlear fibers within the CVN. Audiological evaluation is very important to determine if hearing is present in CC, which indirectly gives an estimate of the cochlear fibers within the CVN. If a behavioral audiometric response or language development is present with hearing aid use, it can be assumed that a meaningful population of cochlear fibers exists and the patient may benefit from a CI. If the CVN cannot be demonstrated with MRI or there is a narrow or long IAC, where the presence of cochlear fibers is questionable, an ABI may be a more appropriate option from the outset. As the postoperative hearing cannot be accurately predicted before CI surgery, it is advisable to counsel the family that contralateral ABI may be necessary in case of limited language development with CI. These cases are regarded as “Possible Indications for an ABI” (Table 1) (5). This decision should be done as early as possible.

### Audiological Findings

These patients usually have profound hearing loss.

### Management

• For CI surgery, the surgical approach is via a transmastoid labyrinthotomy as described by McElveen et al. (6) with a straight (non-modiolar hugging) electrode. This will have a position on the periphery of the CC with better contact with the neural tissue. A pre-curved electrode will have the contacts located medially and may not stimulate the periphery of the CC efficiently. There is a variation in the size of CC. Therefore, correct length of the electrode should be estimated before surgery. The length of the electrode can be calculated using the formula 2πr, where r is the radius of CC.

• Beltrame et al. (7) described a special electrode for CC. This electrode has a inactive tip which is caught by a hook through another hole. Two labyrinthotomy openings are created in the area of lateral SCC, separated by 3-4 mm. The terminal nonactive part of the electrode array ends with a small ball, which is needed to hook the electrode array. This nonactive part of the implant is pushed into the superior labyrinthotomy until it is seen and hooked using a 0.5 mm hook through the inferior labyrinthotomy. Then the two arms are advanced together to position the array along the inner wall of the cavity through the inferior labyrinthotomy.

• In case of insufficient hearing and language development with CI use, ABI may be indicated on the contralateral side (possible ABI indication).

• If CVN is absent or no IAC is present, ABI is the only option in the first place.

### Cochlear Hypoplasia and Incomplete Partitions

In these two groups of malformations, there is a clear differentiation into a cochlea and vestibule.

### 5- Cochlear Hypoplasia

In this deformity, there is a clear differentiation between cochlea and vestibule. CH represents a group of cochlear malformations where external dimensions are less than those of a normal cochlea with various internal architecture deformities. In smaller cochlea, it is usually difficult to count the number of turns with CT and/or MRI. But the definition “cochlea with 1.5 turns” should be used for hypoplasia (particularly type III), rather than for IP-II cochlea. Four different types of CH have been defined (4):

### Types of Cochlear Hypoplasia

### a- CH-I (Bud-like cochlea)

The cochlea is like a small bud, round or ovoid in shape, arising from the IAC (Figure 6a). Internal architecture is severely deformed; modiolus and interscalar septa cannot be identified.

### b- CH-II (Cystic hypoplastic cochlea)

The cochlea has smaller dimensions with defective modiolus and interscalar septa, but with normal external outline (Figure 6b). There may be complete absence of modiolus creating a wide connection with the IAC, making gusher and misplacement of CI electrode into IAC possible. The vestibular aqueduct may be enlarged and the vestibule may be dilated. They may have recurrent meningitis because of defective stapes footplate.

### c- CH-III (Cochlea with less than 2 turns)

The cochlea has fewer turns (less than 2 turns) with a short modiolus. The overall length of the interscalar septa is reduced. The internal (modiolus, interscalar septa) and external outline are similar to that of a normal cochlea, with fewer number of turns and smaller dimensions (Figure 6c). The vestibule and the SCCs are usually hypoplastic. The CA may be hypoplastic or aplastic.

### d- CH-IV (Cochlea with hypoplastic middle and apical turns)

The cochlea has a normal basal turn, but middle and apical turns are severely hypoplastic and located anterior and medially rather than in their normal central position (Figure 6d). The labyrinthine segment of the facial nerve is usually located anterior to the cochlea rather than in its normal location (8).

### Audiological Findings

These patients may demonstrate the full spectrum of hearing loss. They may have normal, mild or moderate hearing loss which can be rehabilitated with hearing aids only. Also pure conductive hearing loss is not uncommon which may benefit from stapes surgery. In the case of mixed hearing loss the patient benefit from stapes surgery and hearing aids. Profound hearing loss is also possible and can be rehabilated with CI and in the case of CN deficiency; with ABI. In conclusion CH patients represent the most interesting group of IEMs regarding clinical presentation and management.

### Management

Decision making in patients with CH may be challenging. They may present with a range of different thresholds on audiometric testing; mild-profound sensorineural, conductive or mixed hearing loss. Decision making about the amplification options may be difficult, particularly in patients with a hypoplastic cochlear nerve.

• Some cases of CH (particularly CH-III and CH-IV) may have pure conductive or mixed hearing loss where the conductive component is due to stapedial fixation. They may benefit from stapedotomy. This can be done in childhood and can result in better oral language development with or without hearing aid use, depending on the bone conduction levels.

• Patients with mild to moderate SNHL can be habilitated with hearing aids and have near normal language development.

• Majority of CH patients have severe to profound hearing loss where a CI would be a reasonable option, if they have a cochlear nerve. During surgery, facial nerve malposition is to be expected due to associated semicircular abnormalities (particularly lateral SCC). In hypoplastic cochlea, the promontory may not have the usual protuberance and it may be difficult to identify promontory and round window through the facial recess. In these situations, an additional transcanal approach may be necessary to expose the hypoplastic cochlea.

• As the number of turns are smaller with narrower scalae, it is strongly advisable to use thin and shorter electrodes. Thick and long electrodes may not be inserted fully into the cochlea. Particularly important is CH II which has a potential for CSF leakage. FORM 19 (Med El) electrode is developed for these cases. It is 19 mm in length and makes one full turn around the hypoplastic and narrow basal turn in addition to providing effective sealing of the cochleostomy. If a longer electrode is chosen, it cannot be inserted up to the silicon stopper in case of gusher in CH-II.

• Some patients with CH have CA aplasia with cochlear nerve aplasia and thus, an ABI would be the best hearing habilitative option.

• Cochlear nerve deficiency is frequently observed in patients with CH. The best option in these cases is to perform CI in the side with better developed cochlear nerve or better audiological findings. If there is limited hearing and language development with CI, an ABI should be considered for the contralateral side. In certain cases, who are between 2-3 years of age, with barely visible cochlear nerves, CI and ABI can be done simultaneously; this is to avoid the loss of valuable time until ABI surgery if the chance of benefit from CI surgery is low. Simultaneous CI and ABI surgery has been performed in three patients in our department.

### 6- Incomplete Partition of the Cochlea

Incomplete partition anomalies represent a group of cochlear malformations, where there is a clear differentiation between cochlea and vestibule, with normal external dimensions and various internal architecture defects. Incomplete partitions constitute 41% of IEMs according to the database of Hacettepe University Department of Otolaryngology. There are three different types of incomplete partition groups according to the defect in the modiolus and the interscalar septa.

### Types of incomplete partition groups

### a- Incomplete partition type I (IP-I)

This type of incomplete partition anomaly was termed as “cystic cochleovestibular malformation” in 2002 by Sennaroglu and Saatci (9). These represent approximately 20% of IEMs. In this anomaly, there is a clear differentiation between cochlea and vestibule. Cochlea is located in its usual location in the anterolateral part of the fundus of the IAC and lacks the entire modiolus and interscalar septa (Figure 7), giving the appearance of an empty cystic structure. External dimensions (height and length) of an IP-I cochlea are similar to normal cases (10). Cochlea is accompanied by an enlarged, dilated vestibule (Figure 7). Vestibular aqueduct enlargement is very rare. There may be a defect between the IAC and the cochlea due to developmental abnormality of the CA and absence of the modiolus and CSF may completely fill the cochlea.

Recurrent meningitis can occur in IP-I patients even prior to their CI surgery or in their non-operated ear. This is due to defective stapes footplate and CSF filling the cochlea. There is a cystic structure in the stapes footplate which is easily infected during an attack of otitis media. This is very characterstic for IP-I. Spontaneous CSF fistula and recurrent meningitis can be seen although less frequently in CH type II. This is because both IP-I and CH-II have endosteal developmental anomaly leading to defective footplate development (4). It is interesting to note that IP-III cases always have a high volume CSF gusher during CI surgery but meningitis is very rarely reported in these patients (1,11). This is most likely due to the fact that the stapes footplate is normally developed, because in IP-III pathology is in the outer two layers of the otic capsule and endosteum is normal. Therefore, a defect in the footplate in IP-III is very unlikely.

All patients with IP-I and recurrent meningitis who have normal tympanic membranes but fluid filling the middle ear and mastoid should have an exploration of the middle ear with special attention to the stapes footplate. Endaural incision was used, and after entering the middle ear, usually a large cyst coming from a defect at the stapes footplate was discovered and excised. After excising the cyst, a defect at the oval window was observed, and CSF gusher was encountered. If the defect is small it sould be enlarged with a 0.6 mm dimond bur for easier manipulation during plugging of the defect. It is also very important to keep stapes suprastructure and the ossicles intact in order to stabilize the fascia in place.

### Management

• Majority of IP-I patients have severe to profound SNHL. They are almost always candidates for CI.

• Size of the cochlea is normal. Therefore, straight electrode about 25 mm is preferred. Modiolar hugging electrode should not be used. FORM 24 (Med El) is developed for these cases. The length is 24 mm which makes a full turn around the basal turn. The conical stopper is used to stop CSF leakage around electrode (12). It is passed through a 2x2 mm fascia and they are inserted together. Silicon stopper pushes and stabilizes the fascia into the cochleostomy. It also keeps it in place.

Digisonic Classic and Digisonic Evo electrodes (Oticon) are also have a silicon stopper which might be useful for controlling gusher.

• In case of gusher, it is most important to stop the CSF leakage from the cochleostomy. The surgeon should not leave operation room without controlling the leakage. After controlling the leakage, subtotal petrosectomy can be done as an additional measure. The benefit of subtotal petrosectomy in CI is to seal the middle ear space from the nasal passage. But if the leakage is not controlled and continues around the electrode, subtotal petrosectomy should not be performed. Most important issue is controlling the leakage from the cochleostomy.

• As it is possible to have CN aplasia in IP-I, some patients may not be a candidate for CI surgery. Therefore, an ABI is indicated in IP-I patients with aplastic CN. Four patients with IP-I and an aplastic CN have received ABI in our department.

• As in CC, an ABI may be indicated on the contralateral side in case of insufficient progress with CI as a possible indication for ABI (5).

### b-Incomplete partition type II (IP-II)

In IP-II, the apical part of the modiolus is defective (Figure 7b). This anomaly was originally described by Carlo Mondini and together with a minimally dilated vestibule and an enlarged vestibular aqueduct (EVA) (Figure 7c) constitute the triad of the Mondini deformity. The term “Mondini” should be used only if the above mentioned triad of malformations is present (1,9,11,13). The apical part of the modiolus and the corresponding interscalar septa are defective, giving the apex of the cochlea a cystic appearance due to the confluence of middle and apical turns. The external dimensions of the cochlea (height and diameter) are similar to that seen in normal cases (10). Therefore, it is not correct to define this anomaly as a cochlea with 1.5 turns (10). The term “cochlea with 1.5 turns” should be used only for CH.

A recent study on histopathology demonstrated that modiolar defects may be due to high CSF pressure transmission into the inner ear as a result of EVA (14). An enlarged endolymphatic sac and duct appears to be the only genetic abnormality that is causing the other abnormalities allowing high CSF pressure to be transmitted into the inner ear. This results in a mild dilatation in the walls of the vestibule. However, no hydropic changes were observed in the endolymphatic space. Depending on the severity and timing of the insult, the pathology may stay at this stage and cause EVA only, or with the transmission of CSF pressure into the cochlea, it may cause a spectrum of anomalies ranging from scala vestibuli dilatation, scala communis, superior (cystic apex), partial, subtotal and in some cases complete modiolar defects. The high pressure in the SV causes bulging of the ISS upwards. This is a constant finding in all cases, showing that cochlear pathology may be the result of high pressure in the SV and that it happened during the developmental phase, otherwise high pressure would have fractured the osseous spiral lamina. If there is higher pressure, it is natural to expect more destruction at the upper, and possibly the lower part of the modiolus. During CI surgery in IP-II, pulsation observed at the round window is due to third window defect of EVA transmitting CSF pressure into the cochlea. CSF oozing and gusher sometimes observed in CI surgery in IP-II are due to modiolar defects occurring as a result of high CSF pressure transmission.

### Audiological Findings

These patients do not have a characteristic hearing level, as their audiometric threshold testing varies from normal to profound. The hearing loss can be symmetric or asymmetric, but it is usually progressive. It is also possible to have sudden SNHL. In addition, there is an air bone gap particularly at low frequencies. Tympanometry is normal in the absence of otitis media and acoustic reflexes are generally present. Air bone gap in these children is likely to be due to a “third window” effect from the EVA, and may resemble the audiometric findings of superior canal dehiscence syndrome.

### Management

• At a young age, these patients may have near normal hearing and may not require amplification initially. With fluctuations and progressive hearing loss, they become candidates for hearing aid.

• Usually the progression in hearing loss continues, ultimately creating a need for CI at some point in the future. In our department we have also followed IP-II cases with profound SNHL since birth necessitating CI surgery at 1 year of age. High pulsating CSF pressure may be responsible for the progression of hearing loss. A role for head trauma has been suggested, and these patients are advised to wear helmets when playing sports and avoiding contact sports completely. As basal part of the modiolus is normal all kinds of electrodes can be used during surgery. Six of the 77 patients with IP-II had severe gusher during CI surgery. Oozing is also common in these patients. Therefore, electrode with silicon stopper is advisable in IP-II. FORM 24 makes one full turn around the basal turn and controls CSF leakage around the electrode. Digisonic Classic and Digisonic Evo electrodes (Oticon) are also have a silicon stopper which might be useful for controlling gusher.

• Stapedotomy should not be performed in these cases as air-bone gap is most probably due to third window effect of EVA.

• As all cases of IP-II have cochlear nerve, ABI is not indicated.

### c- Incomplete partition type III (IP-III)

Cochlea in IP-III has interscalar septa but the modiolus is completely absent (Figure 7d). IP-III cochlear malformation is the type of anomaly present in X-linked deafness, which was described by Nance et al. (15) for the first time in 1971. Phelps et al. (16) described the HRCT findings associated with this condition for the first time, and this characteristic deformity was included under the category of incomplete partition deformities for the first time by Sennaroglu et al. (17) in 2006.

This anomaly is the rarest form of incomplete partition cases. IP-III constitutes 2% of the IEMs in the database in Hacettepe University Department of Otolaryngology.

In IP-III cochlear otic capsule around the membranous labyrinth is thinner when compared to that in a normal cochlea. HRCT demonstrates that in IP-III, the otic capsule around the cochlea is thin and follows the outline of the membranous labyrinth as if it is formed by a thick endosteal layer. Instead of the usual three layers, probably the second and third layers are either absent or very thin. Innermost endosteal layer appears to be thickened without enchondral and outer periosteal layers (4).

Phelps et al. (16) reported that there is a bulbous IAC, incomplete separation of the coils of the cochlea from the IAC. Talbot and Wilson (18) later added that the modiolus is absent and there is a more medial origin of the vestibular aqueduct with varying degrees of dilatation. Sennaroglu et al. (19) reported that in this deformity the interscalar septa are present but the modiolus is completely absent. This gives the cochlea a characteristic appearance. From an earlier study, the external dimensions of the cochlea (height and diameter) were found to be similar to the normal cochlea (17), therefore, it is appropriate to include IP-III under the incomplete partition anomalies. In addition, labyrinthine segment of the facial nerve is located almost above the cochlea (20) instead of making a gentle curve around the basal turn on axial sections. The labyrinthine segment of the facial nerve is the most superior structure in the temporal bone. Thin otic capsule around cochlea and labyrinth, consisting of only a thick endosteal layer, may be responsible for this. Tympanic and mastoid segments appear to be in their normal position.

### Audiological Findings

In IP-III there may be mixed type HL or profound SNHL. Conductive component may be due to thin otic capsule. Stapes surgery is contraindicated in this group as it may lead to gusher and further SNHL.

### Management

Mixed hearing loss gives the impression of stapedial fixation. Stapedotomy results in severe gusher and further SNHL, and thus, should be avoided.

• Patients with moderate to severe mixed or SNHL can be managed with hearing aids.

• Patients with severe HL are candidates for CI. Because of the absent modiolous and large defect at the cochlear base, all patients with IP-III have severe gusher during CI surgery and there is a very high chance of electrode misplacement into IAC. The position of the electrode should be checked intraoperatively in all cases of IP-III. Modiolar hugging electrodes are to be avoided in IP-III. FORM 24 electrodes make one full turn around the cochlear base and also control CSF leakage around the electrode. If the interscalar septa are thick they reduce intracochlear volume and a long electrode may be misdirected into IAC. In such a case FORM 19 is advisable. Digisonic Classic and Digisonic Evo electrodes (Oticon) are also have a silicon stopper which might be useful for controlling gusher but they are longer than FORM electrodes, and they might go into the IAC. Spontaneous CSF fistula through the stapes footplate and recurrent meningitis is very rare in IP-III in spite of high volume CSF leak during CI surgery. This is most probably due to normal endosteal development (hence a normal footplate) in IP-III.

• All IP-III cases have excellent cochlear nerves. Therefore, ABI is not indicated in this group of incomplete partitions.

### 7- Enlarged Vestibular Aqueduct (EVA)

This describes the presence of an enlarged vestibular aqueduct (i.e. the midpoint between posterior labyrinth and operculum is larger than 1.5 mm) in the presence of a normal cochlea, vestibule and SCCs (Figure 8a). Difference between EVA and IP-II is that cochlea and vestibule are completely normal on HRCT and MRI.

In a previous study EVA is thought to be responsible for the transmission of CSF pressure into the inner ear causing progressive or sudden SNHL (4). It appears to be due to a genetic defect. But progressive SNHL is a result of a third window phenomenon.

Classically EVA is described when the midpoint between posterior labyrinth and operculum is larger than 1.5 mm on axial sections. In our department, we have made the observation that EVA can be observed in a number of successive axial images. It may therefore, not be correct to evaluate EVA only on axial sections. We have to take vertical dimension of EVA into account as well. More correct definition of EVA may be “vertical and axial width larger than 1.5 mm on the midpoint between labyrinth and operculum” (Figure 8b).

Audiological presentation and management is similar to that of IP-II.

### 8- Cochlear Aperture Abnormalities

The CA, cochlear fossette, or bony cochlear nerve canal transmits the cochlear nerve from the cochlea to IAC. This can be visualized in the mid-modiolar view as well as coronal sections on HRCT (Figure 1a).

The CA is considered hypoplastic (Figure 9a) if the width is less than 1.4 mm (20). The CA is considered to be aplastic when the canal is completely replaced by bone or there is no canal on mid-modiolar view (Figure 9b).

CA abnormalities may be accompanied by a narrow IAC on HRCT (Figure 9c). The IAC is considered narrow if the width of the midpoint of the IAC is smaller than 2.5 mm. Narrow IAC can accompany other malformations or with a normal cochlea. In cases of narrow IAC, MRI should be obtained to demonstrate if CN is normal, aplastic or hypoplastic. Axial and sagittal oblique high T2 weighted images (i.e. CISS, Fiesta etc.) images are necessary for this purpose. In CN aplasia, no nerve can be identified in the anterior inferior part of the IAC.

On axial section CN is followed until modiolus (Figure 9d). On sagittal oblique MR sections, four distinct nerves can be visualized in the IAC (Figure 9e). CA aplasia is typically accompanied by cochlear nerve aplasia. CN may be hypoplastic (Figure 9f, 9g) or aplastic when CA is hypoplastic. CA hypoplasia and aplasia can also be observed in a normal cochlea.

### Audiological Findings

Severe to profound SNHL is usually present. As the cochlea is normal, otoacoustic emissions (OAE) may be present and the child may pass newborn hearing screening if automated ABR is not obtained. Their hearing loss is typically discovered later on in childhood based on the family’s concerns of lack of sound awareness and language development. If the newborn screening protocol involves OAE and automated ABR, this malformation can be diagnosed during infancy. Diagnostic audiological evaluation will reveal profound hearing loss.

### Management

Hearing aids usually do not provide sufficient amplification in patients with CA hypoplasia and aplasia. In patients with bilateral hypoplastic CA with hypoplastic cochlear nerve, hearing aid trial is necessary. If this does not provide adequate functional hearing, these patients usually become candidates for CI. The family should be counseled that if CI does not provide sufficient hearing in terms of auditory perception, contralateral ABI may be necessary to achieve improved audiologic and language outcomes.

In CA aplasia, ABI is indicated as a first-line therapy.

## II- COCHLEAR NERVE ABNORMALITIES

The classification of CVN is also important in the management of IEMs.

### 1- Normal cochlear nerve (CN)

It is important to trace the CN until it enters the cochlea on lower axial sections passing through the IAC (Figure 9d). On parasaggital sections, there is a separate CN located in the anterior inferior part of the IAC, entering the cochlea (Figure 9e). The size of the cochlear nerve is similar in size when compared with the CN on the contralateral normal side. According to Casselman et al. (21) on parasaggital view the size of the CN is similar or slightly larger than the ipsilateral FN.

### 2- Hypoplastic CN

There is a separate CN but the size is less than the contralateral normal CN or ipsilateral normal facial nerve (Figure 9f, 9g).

### 3- Absent CN

There is no nerve in the anteroinferior part of the IAC (Figure 9h, 9ı). This is definitely present in cochlear aplasia. It can also be seen in CA hypoplasia and aplasia.

### 4- Normal CVN

Normally cochlear and vestibular nerves originate at the brainstem together forming the CVN. CVN then separates into CN and superior and inferior vestibular nerves in the IAC. In cases of CC CVN enters the cavity without separating into individual nerves (Figure 9j). With radiological precision at the present time, it is impossible to determine the cochlear fiber content in the CVN but if the size is 1.5-2 times as much as the ipsilateral FN or similar to contralateral normal CVN it can be accepted as normal.

### 5- Hypoplastic CVN

If CVN is smaller than contralateral CVN or ipsilateral FN, it can be accepted as hypoplastic (Figure 9k). CVN hypoplasia is particularly important in CC.

### 6- Absent CVN

In case of Michel deformity with absent IAC, CVN is also absent (Figure 9l). Only facial nerve can be identified.

### Audiological Findings

Severe to profound SNHL is usually present. As the cochlea is normal, OAE may be present and the child may pass newborn hearing screening if automated ABR is not obtained. The amount of cochlear nerve fibers determine the hearing level and management strategy. However, for the present time there’s no spesific auditory profile characteristics for different CVN subgroups. It’s advisable to evaluate the candidate with the full audiological test battery.

### Management

After a hearing aid trial, initial approach is a trial with CI. If the progress of the patient is insufficient, it is advisable to progress with ABI. This procedure is suitable if the initial CI surgery was done around the age of 6 months; ABI can then be done between 1-1.5 years old with acceptable language development. However if the patient comes in around the age of 2, the time delay between two surgeries increases hence the outcome of ABI decreases. Therefore, simultaneous CI and ABI maybe a better option in selected cases. In our department simultanous CI and ABI have been performed in three such cases.

## CONCLUSION

IEMs is a special group of patients. Unfortunately, until recent years the term “Mondini” has been used to describe many different anomalies. There are variety of IEMs and they all present in a different way. As can be seen easily each group has different characteristics in terms of presentation, radiology, hearing and surgical findings. Proper classification is very important in the management of IEMs. If the anomaly is correctly classified, appropriate treatment can be decided more correctly. Finally, proper classification also will create a common language around the world, where clinicians can understand each other better.

## Figures and Tables

**Table 1:**
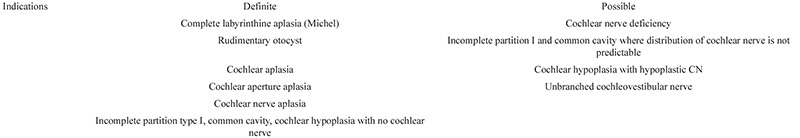
Definite and possible indications for auditory brainstem implantation

**Table 2:**
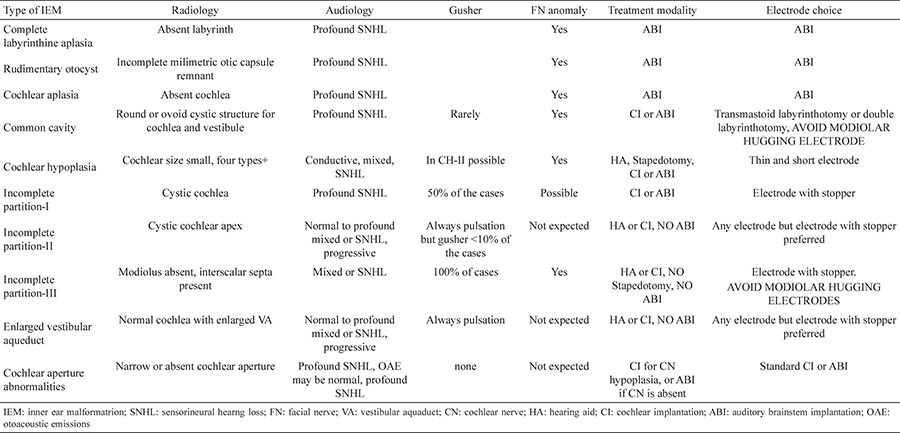
Characteristics of the inner ear malformations

**FIG. 1a.**
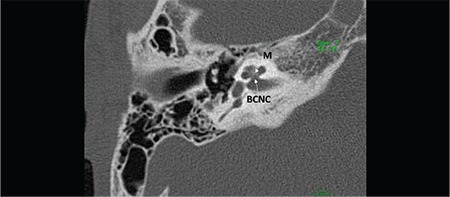
Mid-modiolar view demonstrating modiolus (M) and cochlear aperture, bony canal for cochlear nerve.

**FIG. 1b.**
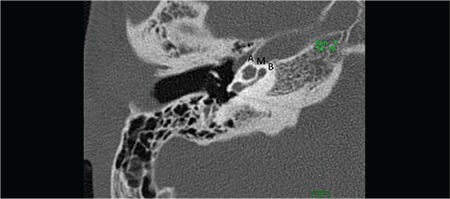
Section passing through round window niche (Figure 1b), showing basal (B), middle (M), and apical (A) cochlear turns.

**FIG. 1c.**
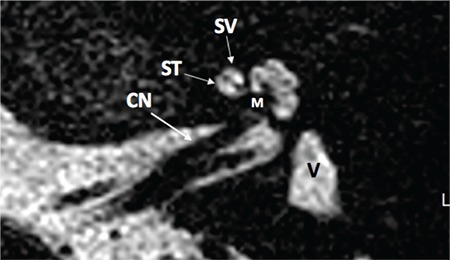
Axial T2 weighted magnetic resonance image showing cochlear nerve (CN), scala tympani (ST), scala vestibuli (SV), modiolus (M) and vestibule (V).

**FIG. 4a.**
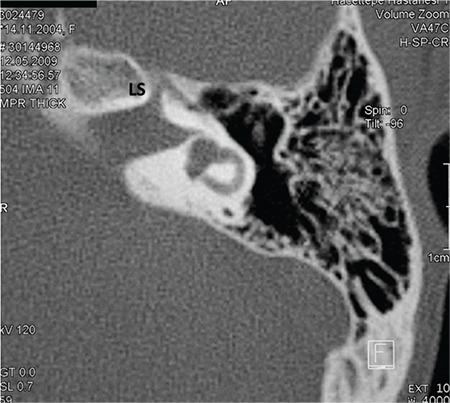
Cochlear aplasia with normal labyrinth: vestibule and semicircular canals are normal (location and development), Labyrinthine segment of the facial nerve (LS) is anteriorly dislocated.

**FIG. 4b.**
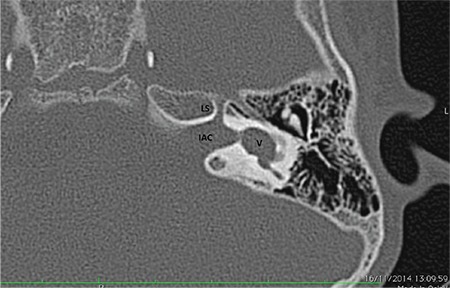
Cochlear aplasia with a dilated vestibule: vestibule (V) and semicircular canals are located normally but there is vestibular dilatation. Labyrinthine segment of the facial nerve (LS) is anteriorly dislocated. IAC: internal auditory canal

**FIG. 6a.**
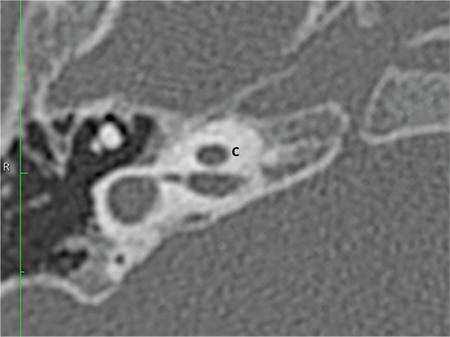
CH-I (Bud-like cochlea); a small bud, round or ovoid in shape, arising from the IAC.

**FIG. 6b.**
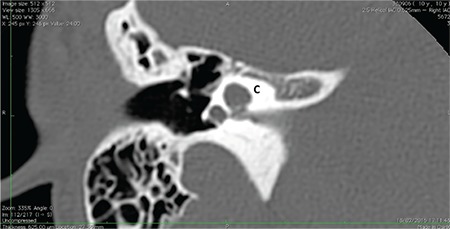
CH-II (Cystic hypoplastic cochlea) cochlea has smaller dimensions with defective modiolus and interscalar septa, but with normal external outline.

**FIG. 6c.**
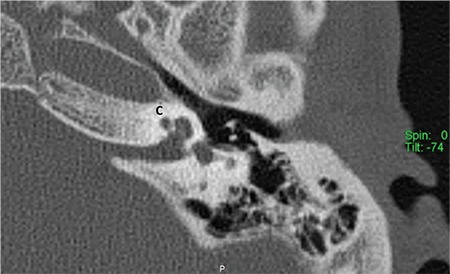
CH-III (Cochlea with less than 2 turns) The cochlea has fewer turns (less than 2 turns) with a short modiolus.

**FIG. 6d.**
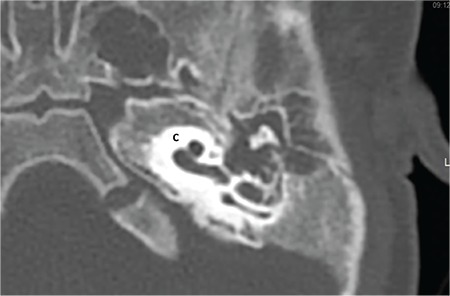
CH-IV (Cochlea with hypoplastic middle and apical turns) cochlea has a normal basal turn, but middle and apical turns are severely hypoplastic.

**FIG. 7a.**
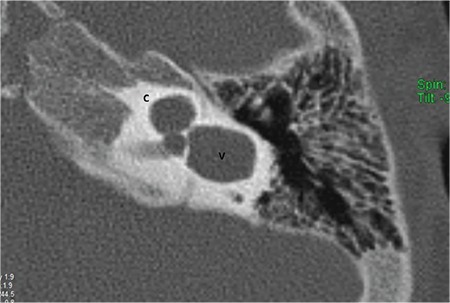
Incomplete partition-I; Cochlea (C) without modiolus and interscalar septa, accompanied by an enlarged, dilated vestibule (V).

**FIG. 7b.**
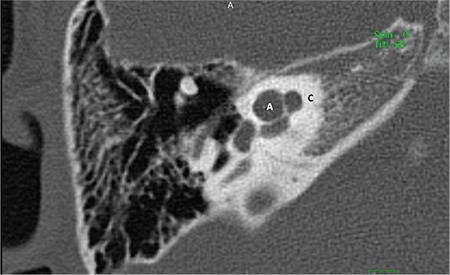
Incomplete partition-II; Cystic apical part (A) of the cochlea (C).

**FIG. 7c.**
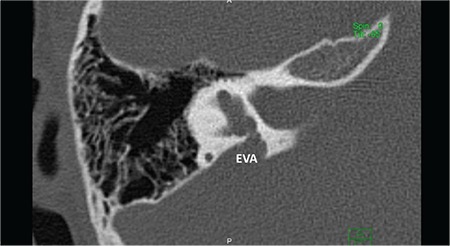
Incomplete partition-II; enlarged vestibular aqueduct (EVA).

**FIG. 7d.**
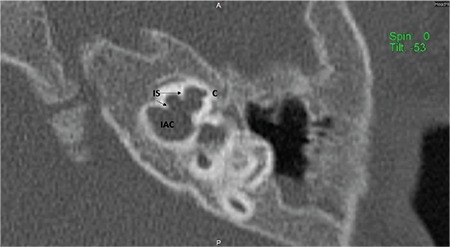
Incomplete partition-III. Cochlea (C) has interscalar septa (IS) but the modiolus is completely absent.

**FIG. 8a.**
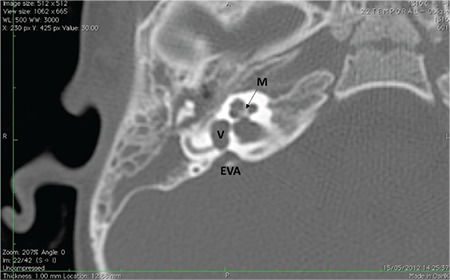
Axial section; Enlarged vestibular aqueduct (EVA) with normal modiolus (M) and normal vestibule (V).

**FIG. 8b.**
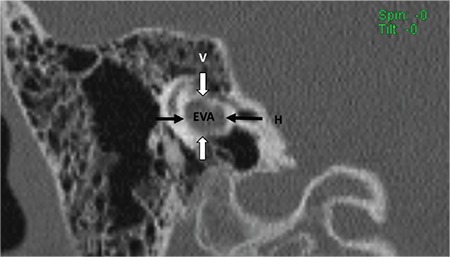
Coronal section; Enlarged vestibular aqueduct (EVA) with vertical dimensions (V white arrow) and horizontal dimensions (H, black arrow).

**FIG. 9a.**
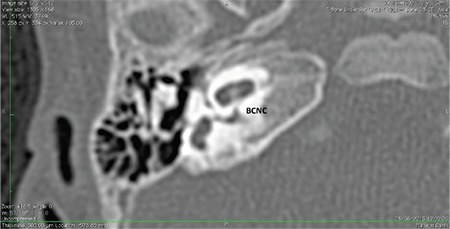
Hypoplastic bony canal for cochlear nerve (BCNC).

**FIG. 9b.**
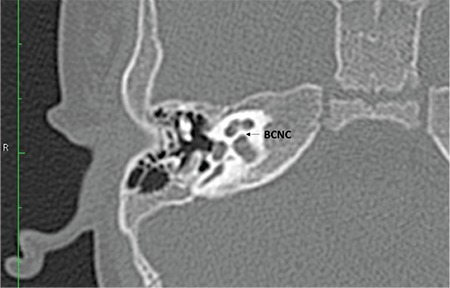
Aplastic bony canal for cochlear nerve (BCNC).

**FIG. 9c.**
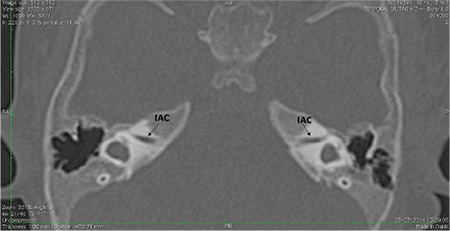
Bilateral narrow internal auditory canal (IAC) accompanying bilateral absent cochlear nerve.

**FIG. 9d.**
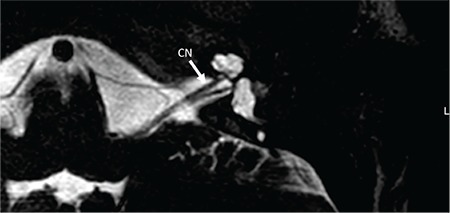
On axial section cochlear nerve (CN) is followed until modiolus.

**FIG. 9e.**
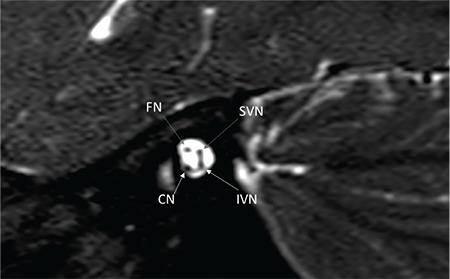
On sagittal oblique MR sections, four distinct nerves can be visualized in the IAC. FN: facial nerve; CN: cochlear nerve; SVN: superior vestibular nerve; IVN: inferior vestibular nerve

**FIG. 9f.**
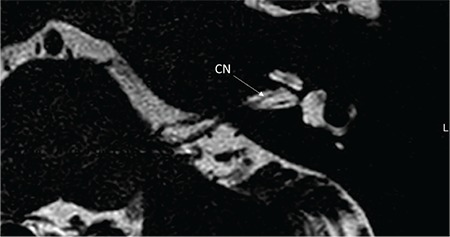
Hypopastic cochlear nerve (CN), axial view.

**FIG. 9g.**
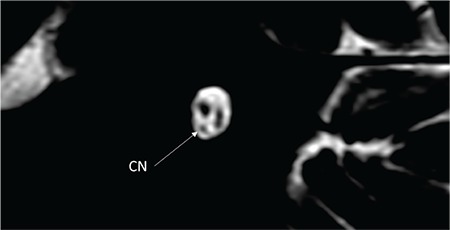
Hypoplastic cochlear nerve (CN), saggital oblique view.

**FIG. 9h.**
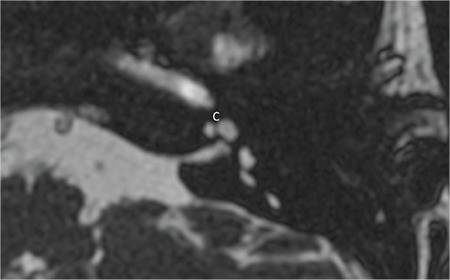
Aplastic cochlear nerve, axial view.

**FIG. 9i.**
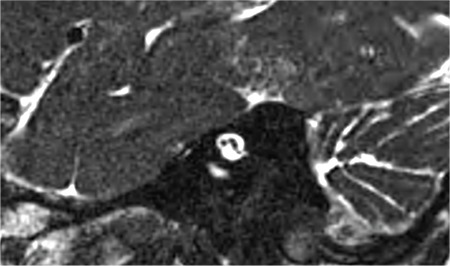
Aplastic cochlear nerve (CN), saggital oblique view.

**FIG. 9j.**
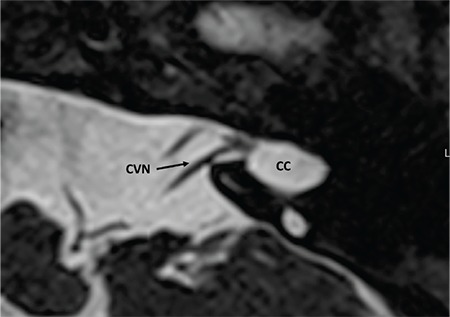
Cochleovestibular nerve (CVN) entering common cavity (CC).

**FIG. 9k.**
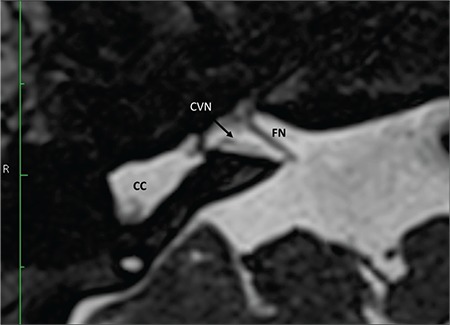
A hypoplastic cochleovestibular nerve (CVN) entering common cavity (CC). Size of the facial nerve (FN) is much larger than the CVN.

**FIG. 9l.**
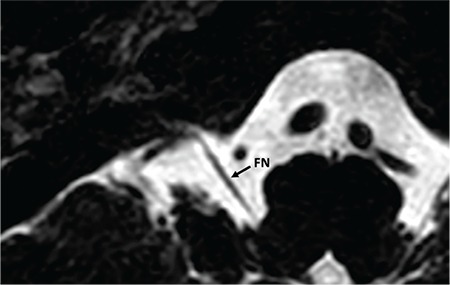
Absent cochleovestibular nerve. In a case of complete labyrinthine aplasia only facial nerve (FN) is present. Cochleovestibular nerve is absent. FN enters the temporal more anteriorly than its usual entry point.
